# Capecitabine May Accelerate Atherosclerosis and Causes Acute Myocardial Infarction in the Left Main Trunk

**DOI:** 10.7759/cureus.39170

**Published:** 2023-05-18

**Authors:** Tetsuo Yamanaka, Tatsuhiko Ishihara, Koutarou Miyata, Yoshimaro Ichinohe, Toru Fukatsu

**Affiliations:** 1 Cardiology, Tokyo Teishin Hospital, Tokyo, JPN; 2 Cardiology, Kanto Central Hospital of the Mutual Aid Association of Public School Teachers, Tokyo, JPN; 3 Cardiology, St. Luke’s International Hospital, Tokyo, JPN

**Keywords:** colon cancer, acute myocardial infarction, atherosclerosis, cardiotoxicity, capecitabine

## Abstract

We report a case of a 59-year-old man who developed acute myocardial infarction which is supposed to be associated with capecitabine administration. At the age of 57 years, the patient underwent a laparoscopic colectomy for sigmoid colon cancer and subsequently received adjuvant chemotherapy with capecitabine. About one year later, he developed an acute myocardial infarction and was treated with percutaneous coronary intervention. He did not demonstrate any coronary risk factors except dyslipidemia, which itself was unlikely to be involved in prominent atherogenesis. Considering the reports so far, we presumed that capecitabine contributed to the progression of atherosclerosis in the present case.

## Introduction

Chemotherapy for malignancies has progressed remarkably and employs a wide variety of agents. Capecitabine, an oral chemotherapy agent classified as a fluoropyrimidine, is metabolized in the liver to 5’-deoxy-5-fluorocytidine by carboxylesterase and to 5’-deoxy-5-fluorouridine by cytidine deaminase. It is finally converted to 5-fluorouracil (5-FU) by thymidine phosphorylase, which is present in high concentrations in tumors. 5-FU exerts its antitumor effect primarily by inhibiting the production of DNA precursors.

In postoperative chemotherapy for colon cancer, capecitabine has been shown to be effective and non-inferior to conventional treatment [1]. Therefore, it has been used widely in recent years. However, the widespread use of capecitabine has led to multiple reports of cardiotoxicity, including cases of coronary artery spasm or myocardial infarction due to atherosclerosis [2-15].

## Case presentation

A 59-year-old man underwent a medical checkup at the age of 58 years. The examination revealed that his low-density lipoprotein cholesterol (LDL-C) level was 150 mg/dL, and he was followed up without oral medication. He had no history of hypertension, diabetes, smoking, or family history of cardiovascular disease. He has never had tests to assess atherosclerosis, such as a carotid ultrasound. His height, body weight, and body mass index were 165 cm, 52.7 kg, and 19.7 kg/m^2^, respectively, at the age of 58 years.

At the age of 57 years, the patient underwent laparoscopic sigmoid colectomy for sigmoid colon cancer (stage IIIb).

Subsequently, adjuvant chemotherapy with 4200 mg/day capecitabine (2100 mg twice a day) was started. He underwent four cycles with each cycle consisting of capecitabine administration for 14 consecutive days, followed by a seven-day rest period. He did not exhibit cardiotoxicity or any other severe adverse events. After four cycles of adjuvant chemotherapy, the patient exhibited progress without any evident recurrence of colon cancer and did not perceive any chest pain following the treatment.

**Figure 1:**
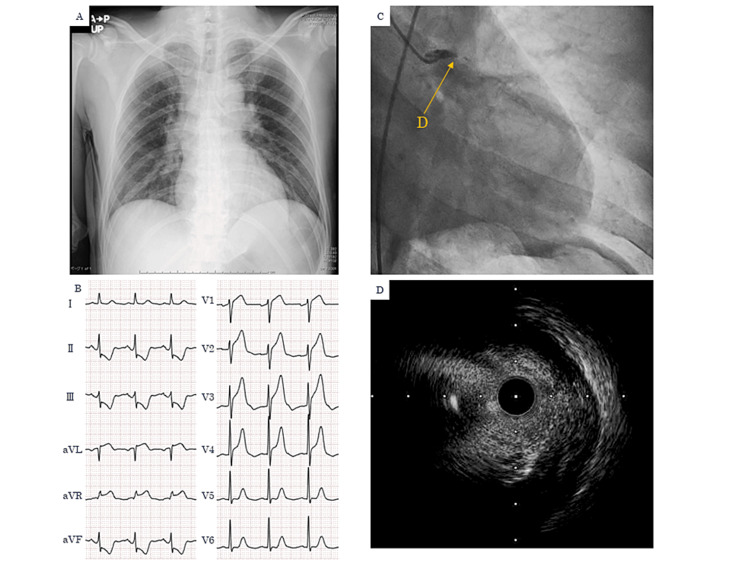
(A) Chest X-rays did not show any evidence of cardiac shadow enlargement, mediastinal widening, or abnormal pulmonary field findings. (B) A 12-lead electrocardiogram revealed ST-segment elevation in leads aVL, aVR, and V1−V4 as well as ST-segment depression in leads II, III, and aVF. (C) Emergency coronary angiography revealed that LMT was occluded. (D) IVUS revealed that low-attenuation plaque was distributed through more than half of the LMT lesion. LMT: left main trunk, IVUS: intravascular ultrasound

About 10 months after the completion of chemotherapy, one hour before visiting our hospital, the patient experienced sudden chest pain at rest. Persistent chest pain prompted him to visit our hospital. On arrival, he was lucid, his blood pressure was 92/62 mmHg, his pulse rate was 67 beats per minute, and his transcutaneous oxygen saturation was 100% (room air). Chest pain was persistent after arrival. Auscultation did not reveal any evident abnormalities in heart sounds or breathing sounds. Chest X-rays did not show any evidence of cardiac shadow enlargement, mediastinal widening, or abnormal pulmonary field findings (Figure 1A). A 12-lead electrocardiogram revealed ST-segment elevation in leads aVL, aVR, and V1−V4 as well as ST-segment depression in leads II, III, and aVF (Figure 1B). Transthoracic echocardiography revealed asynergy in the anterolateral, anterior, and anteroseptal walls of the left ventricle. The patient was diagnosed with acute ST-elevation myocardial infarction and was immediately administered two 100 mg acetylsalicylic acid tablets. His chest pain worsened during the transfer to the catheterization laboratory. Upon arrival at the catheterization laboratory, his blood pressure had deteriorated to 70/50 mmHg. Emergency coronary angiography revealed that his left main trunk (LMT) was occluded (Figure 1C). After the administration of 20 mg prasugrel (Daiichi Sankyo, Tokyo, Japan), he underwent percutaneous coronary intervention (PCI) following emergency intra-aortic balloon pump placement. After the thrombus was aspirated using Thrombuster GR (Kaneka Medical Products, Osaka, Japan), thrombolysis in myocardial infarction (TIMI) grade 3 flow was achieved. Intravascular ultrasound (IVUS) revealed that low-attenuation plaque was distributed through more than half of the LMT lesion, suggesting that these plaques were extremely vulnerable (Figure 1D).

**Figure 2:**
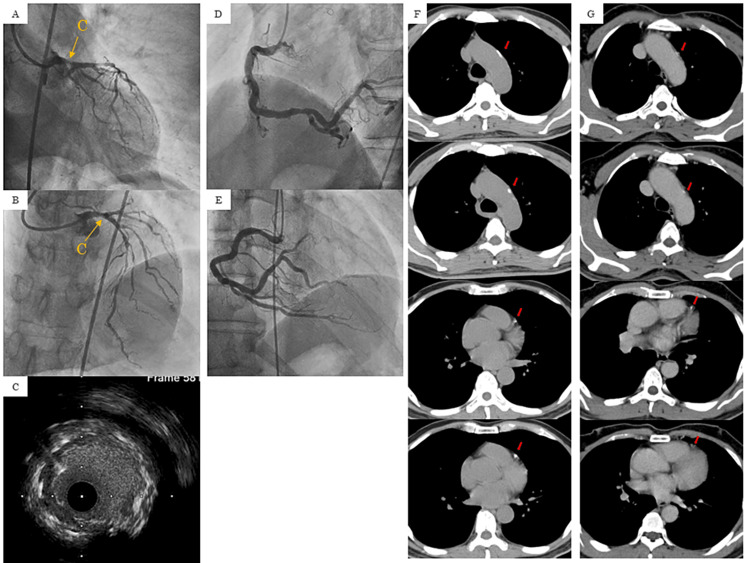
(A, B, C) When a drug-eluting stent was deployed into the LMT to LAD, TIMI grade 3 flow was obtained. Diffuse advanced stenosis was observed in LAD. Thrombus formation in a part of the stent was observed in angiography (yellow arrow) and IVUS. (D, E) Right coronary angiography did not reveal significant stenosis. (F, G) On day three, CT showed exacerbated findings of calcification in the LAD and aorta compared to before capecitabine was administered (red arrow). (C) IVUS findings, (F) on day 3, (G) before capecitabine was administered. Taken in the prone position. LMT: left main trunk, LAD: left anterior descending artery, TIMI: thrombolysis in myocardial infarction, IVUS: intravascular ultrasound, CT: computed tomography

TIMI grade 3 flow was achieved with the deployment of a 3.5 × 18 mm Resolute Onyx stent (Medtronic, MN, USA) from the left anterior descending artery (LAD) into the LMT. However, soon afterward, a thrombus was formed in a part of the stent (Figure 2A, 2B, 2C). The patient was administered 60,000 units of urokinase twice, after which thrombus formation ceased to increase. Although diffuse advanced stenosis (90%) was observed in LAD, the flow score was TIMI grade 3, and his blood pressure also improved to 102/70 mmHg. After that, the right coronary artery angiography was performed, which did not reveal significant stenosis (Figure 2D, 2E). Therefore, the operation was concluded. Door-to-balloon time was 73 minutes. The patient was subsequently managed in the intensive care unit, where 100 mg aspirin, 3.75 mg prasugrel, 10 mg rosuvastatin, and 1.25 mg carvedilol were administered in addition to unfractionated heparin (15,000 units/day). He subsequently exhibited progress with a peak creatine kinase (CK)/CK-MB of 536/65 IU/L and no evident complications. On day three, CT showed exacerbated findings of calcification in the LAD and aorta compared to CT at the age of 57 (Figure 2F, 2G).

**Figure 3:**
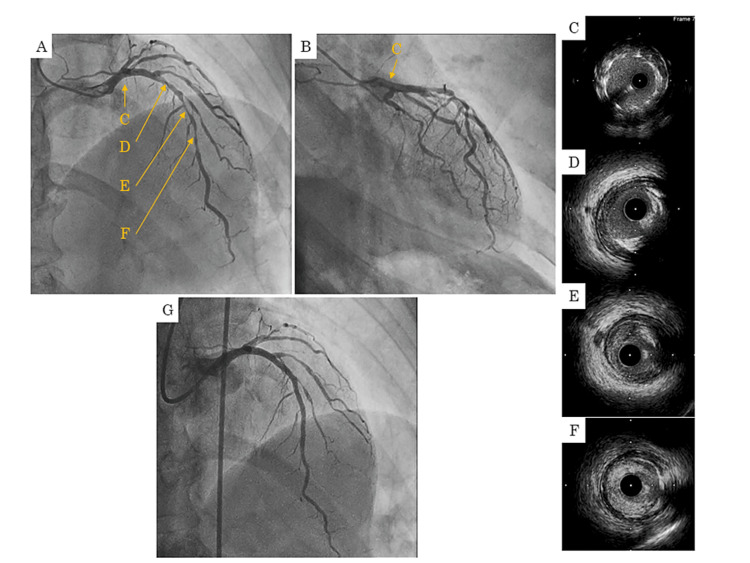
(A, B, C) On day 17, the thrombus in the LMT disappeared according to angiography and IVUS findings. (D, E, F) According to IVUS findings, the lesion in #6-7 primarily consisted of a mixture of fibro-fatty and fibrous plaque with some calcified plaque. (G) Deployment of drug-eluting stent into the lesion resulted in favorable dilation without evident complications. (C, D, E, F) IVUS findings LMT: left main trunk, IVUS: intravascular ultrasound

On day four, 1.25 mg of enalapril was initiated. On day 17, the patient underwent a coronary angiogram, which revealed that the thrombus in the LMT had disappeared according to angiography and IVUS findings (Figure 3A, 3B, 3C). The patient then underwent PCI for the LAD. According to IVUS findings, the lesion in #6-7 primarily consisted of a mixture of fibro-fatty and fibrous plaque with some calcified plaque (Figure 3D, 3E, 3F). Deployment of Resolute Onyx stents (sized 3.0 × 22 mm and 2.5 × 38 mm) (Medtronic, Minnesota, USA) into the lesion resulted in favorable dilation without evident complications, and the operation was concluded (Figure 3G). The patient exhibited progress without any complications and was discharged on day 25. Currently, at 24 months following the treatment, his progress is favorable.

## Discussion

Capecitabine has been used widely in recent years. However, the widespread use of capecitabine has led to multiple reports of cardiotoxicity. Polk et al. [3] conducted a systematic review of 30 eligible studies (one meta-analysis of four randomized controlled trials, 18 prospective studies, and 11 retrospective studies) and reported symptomatic cardiotoxicity in 3-35% of the patients treated with capecitabine. The most common symptom was chest pain (0-18.6%), followed by palpitations (0-23.1%), dyspnea (0-7.6%), and hypotension (0-6%). Severe clinical events such as myocardial infarction, cardiogenic shock, and cardiac arrest occurred in 0-2% of patients. Mortality rates ranged from 0% to 8%. However, this systematic review did not provide a detailed assessment of the coronary artery in individual cases. Detailed case reports of capecitabine-induced coronary artery disease are limited. These reports wherein the coronary artery did not demonstrate organic stenosis and coronary artery vasospasm was finally diagnosed [4-15]. In addition, we found only one case, which presented with myocardial infarction following atherosclerotic changes in the coronary artery [2].

In the present case, since the patient demonstrated dyslipidemia as a coronary risk factor, we could not exclude the possibility that dyslipidemia alone caused atherosclerotic changes in the coronary arteries. Therefore, we investigated the lipid profile in this case before the initiation of statin. Direct LDL-C, high-density lipoprotein cholesterol (HDL-C), and triglyceride levels in our patient were 150 mg/dL, 40 mg/dL, and 120 mg/dL, respectively. He demonstrated a slightly low apolipoprotein A1 level of 92 mg/dL (normal range: 95-180 mg/dL). On the other hand, his apolipoprotein B100 level was 86 mg/dL (normal range: 95-180 mg/dL), which suggested that LDL particle size was not small, and his LDL-C possibly consisted mostly of large buoyant LDL-C. Apolipoprotein C-II and remnant lipoprotein cholesterol levels were normal (2.6 mg/dL [normal range: 1.8-4.6 mg/dL] and 2.1 mg/dL [normal range ≤ 7.5 mg/dL], respectively). Apolipoprotein C-III is a component of remnant lipoproteins, which are highly atherogenic. Loss-of-function mutations in the APOC3 gene, which codes for apolipoprotein C-III, are considered to be associated with a reduced risk of ischemic heart disease [16]. In the present case, the patient’s apolipoprotein C-III level was 4.9 mg/dL (normal range: 5.8-10 mg/dL), which was lower than the normal range. Hence, it was not a risk factor for ischemic heart disease. Lipoprotein(a), a lipid that is composed of LDL constituents apolipoprotein B-100 and apolipoprotein(a) stabilized by disulfide bonds, is considered an independent risk factor for atherosclerosis [17]. Lipoprotein(a) level in our patient was 14 mg/dL (normal range: ≤ 30 mg/dL), which was not high. Based on these findings, dyslipidemia was unlikely to be an extremely high-risk factor for atherosclerosis in our patient. Our patient did not have hypertension, diabetes, a family history of cardiovascular disease, obesity, or a smoking history which are generally considered risk factors for atherosclerosis. In addition, dyslipidemia itself was unlikely to be involved in prominent atherogenesis, as discussed earlier. Therefore, it is unlikely that only the type of dyslipidemia observed in the present case could result in acute coronary syndrome induced by the rupture of vulnerable plaque with low attenuation in the LMT or diffuse advanced stenosis in the left anterior descending coronary artery. Furthermore, atherosclerosis progressed after the administration of capecitabine compared to before the administration on CT. Thus, we presumed that capecitabine contributed to the progression of atherosclerosis in the present case.

Capecitabine is reported to be a risk factor for atherosclerosis, and endothelin-1 (ET-1) is believed to be involved in the mechanism for this risk [6,9]. ET-1 enhances the growth of vascular smooth muscle cells as well as the expression of various growth factors, biologically active substances, and extracellular matrix proteins. It also enhances the expression of the chemoattractant macrophage chemotactic protein-1 and endothelial cell adhesion molecules such as intercellular adhesion molecule-1, vascular cell adhesion molecule-1, and E-selectin. ET-1 is believed to facilitate atherogenesis by promoting the adhesion of macrophages and other monocytes to endothelial cells and their uptake by endothelial cells [18]. 5-FU, which is a metabolite of capecitabine, is believed to promote the production and release of ET-1 from endothelial cells [6,9,19], presumably leading to the progression of atherogenesis. Thymidine phosphorylase activity is also believed to be enhanced in atherosclerotic plaque [20]. This enhanced activity is considered to be synergistically related to the progression of atherosclerosis along with an increased concentration of 5-FU in plaque [9]. However, there are no reports that prove a direct relationship between capecitabine and the acceleration of atherosclerosis, and it is considered necessary to accumulate cases in the future.

Cardiotoxicity is likely to occur 2-3 days after the initiation of capecitabine according to Kuppens et al. [15], and Polk et al. [3] reported that the first occurrence of cardiotoxicity was in the first cycle in 11 patients (50%), in the second cycle in four patients (18%), in the third cycle in three patients (14%), and in the fourth cycle in one patient (4.5%), while three patients (14%) demonstrated late cardiotoxicity (eighth, ninth, and 12th cycle). Thus, early cardiotoxicity was observed in most of the patients. However, Tolga et al. [2] reported a case in which myocardial infarction resulted from atherosclerotic changes. It occurred during chemotherapy three months after the initiation of capecitabine. In our patient, myocardial infarction occurred approximately 13 months after capecitabine initiation and at 10 months after its termination. Unlike that of coronary vasospasm, the onset of capecitabine-induced atherosclerotic changes was evidently late. We presume that this late onset was possibly attributed to the time required for the progression of atherosclerosis.

There are only two reported cases of acute coronary syndrome resulting from capecitabine-induced atherosclerotic changes including the present case. We speculate that the reason for the dearth of case reports might be the lack of recognition of the possibility that capecitabine enhances atherosclerosis and causes coronary artery disease over the course of several months.

## Conclusions

In conclusion, findings from the present case suggest that capecitabine must be used with caution, as it may enhance atherosclerotic changes that can result in acute coronary syndrome. There might be more numbers of cases than the reported cases, and more such cases need to be accumulated in the future.

## References

[1] Twelves C, Wong A, Nowacki MP (2005). Capecitabine as adjuvant treatment for stage III colon cancer. N Engl J Med.

[2] Güvenç TS, Celiker E, Ozcan KS, Ilhan E, Eren M (2012). Acute myocardial infarction after capecitabine treatment: not always vasospasm is responsible. Chin Med J (Engl).

[3] Polk A, Vaage-Nilsen M, Vistisen K, Nielsen DL (2013). Cardiotoxicity in cancer patients treated with 5-fluorouracil or capecitabine: a systematic review of incidence, manifestations and predisposing factors. Cancer Treat Rev.

[4] Cardinale D, Colombo A, Colombo N (2006). Acute coronary syndrome induced by oral capecitabine. Can J Cardiol.

[5] Coughlin S, Das S, Lee J, Cooper J (2008). Capecitabine induced vasospastic angina. Int J Cardiol.

[6] Tsiamis E, Synetos A, Stefanadis C (2011). Capecitabine may induce coronary artery vasospasm. Hellenic J Cardiol.

[7] Frickhofen N, Beck FJ, Jung B, Fuhr HG, Andrasch H, Sigmund M (2002). Capecitabine can induce acute coronary syndrome similar to 5-fluorouracil. Ann Oncol.

[8] Schnetzler B, Popova N, Collao Lamb C, Sappino AP (2001). Coronary spasm induced by capecitabine. Ann Oncol.

[9] Henry D, Rudzik F, Butts A, Mathew A (2016). Capecitabine-induced coronary vasospasm. Case Rep Oncol.

[10] Sestito A, Sgueglia GA, Pozzo C, Cassano A, Barone C, Crea F, Lanza GA (2006). Coronary artery spasm induced by capecitabine. J Cardiovasc Med (Hagerstown).

[11] Rizvi AA, Schauer P, Owlia D, Kallal JE (2004). Capecitabine-induced coronary vasospasm--a case report. Angiology.

[12] Wijesinghe N, Thompson PI, McAlister H (2006). Acute coronary syndrome induced by capecitabine therapy. Heart Lung Circ.

[13] Farina A, Malafronte C, Valsecchi MA, Achilli F (2009). Capecitabine-induced cardiotoxicity: when to suspect? How to manage? A case report. J Cardiovasc Med (Hagerstown).

[14] Shah NR, Shah A, Rather A (2012). Ventricular fibrillation as a likely consequence of capecitabine-induced coronary vasospasm. J Oncol Pharm Pract.

[15] Kuppens IE, Boot H, Beijnen JH, Schellens JH, Labadie J (2004). Capecitabine induces severe angina-like chest pain. Ann Intern Med.

[16] Crosby J, Peloso GM, Auer PL (2014). Loss-of-function mutations in APOC3, triglycerides, and coronary disease. N Engl J Med.

[17] Tsimikas S, Fazio S, Ferdinand KC (2018). NHLBI working group recommendations to reduce lipoprotein(a)-mediated risk of cardiovascular disease and aortic stenosis. J Am Coll Cardiol.

[18] Zouki C, Baron C, Fournier A, Filep JG (1999). Endothelin-1 enhances neutrophil adhesion to human coronary artery endothelial cells: role of ET(A) receptors and platelet-activating factor. Br J Pharmacol.

[19] Porta C, Moroni M, Ferrari S, Nastasi G (1998). Endothelin-1 and 5-fluorouracil-induced cardiotoxicity. Neoplasma.

[20] Boyle JJ, Wilson B, Bicknell R, Harrower S, Weissberg PL, Fan TP (2000). Expression of angiogenic factor thymidine phosphorylase and angiogenesis in human atherosclerosis. J Pathol.

